# Structured early childhood education exposure and childhood cognition – Evidence from an Indian birth cohort

**DOI:** 10.1038/s41598-024-63861-8

**Published:** 2024-06-05

**Authors:** Beena Koshy, Manikandan Srinivasan, Rangan Srinivasaraghavan, Reeba Roshan, Venkata Raghava Mohan, Karthikeyan Ramanujam, Sushil John, Gagandeep Kang

**Affiliations:** 1 Developmental Paediatrics Unit, Christian Medical College, Vellore, 632004 India; 2 Wellcome Research Unit, Christian Medical College, Vellore, 632004 India; 3 Community Health, Christian Medical College, Vellore, 632004 India; 4 Low Cost Effective Care Unit, Christian Medical College, Vellore, 632004 India

**Keywords:** Early childhood education, Children, Preschool, Learning, Cognition, Paediatrics, Paediatric research, Health policy

## Abstract

Experiences in early childhood form the bedrock of future human potential. In impoverished settings, structured early childhood education (ECE) in preschool years can augment overall childhood and later human abilities. The current study evaluates preschool learning exposure and childhood cognition, using longitudinal follow-up of a community-based birth cohort in Vellore, south India. The birth cohort study site in Vellore recruited 251 newborns between 2010 and 2012 from dense urban settlements and further followed up into childhood. Preschool enrolment details were obtained from parents. Childhood cognition was assessed by Weschler’s preschool primary scale of intelligence (WPPSI) and Malin’s intelligence scale for Indian Children (MISIC) at 5 and 9 years of age respectively. Bivariate and multivariate regression analyses were performed with adjustments for socio-economic status (SES), maternal education, stunting status and home environment. Out of 251 new-borns recruited into the MAL-ED birth cohort, 212 (84.46%) and 205 (81.7%) children were available for the 5 year and 9 year follow-up respectively. At 5 years, structured ECE of 18 to 24 months duration was significantly associated with higher cognition scores, with the highest increase in processing speed [β: 19.55 (11.26–27.77)], followed by full-scale intelligence [β: 6.75 (2.96–10.550)], even after adjustments for SES, maternal cognition, home factors and early childhood stunting status. Similarly adjusted analysis at 9 years showed that children who attended 1.5–2 years of structured ECE persisted to have higher cognition, especially in the performance domain [β: 8.82 (2.60–15.03)], followed by the full-scale intelligence [β: 7.24 (2.52–11.90)]. Follow-up of an Indian birth cohort showed that structured ECE exposure was associated with better school entry cognition as well as mid-childhood cognition. Strengthening ECE through a multi-pronged approach could facilitate to maximize cognitive potential of human capital.

## Introduction

Cognition or intelligence can be defined as a ‘combination of multiple abilities in a child’ as proposed by Howard Gardner^1^. The first 1000 days of life starting from the antenatal period in the mother’s womb, to the perinatal period and the first 2 years of life, is crucial for child’s cognitive development, as maximum brain development and maturation happen during this period^2^. Factors such as early childhood malnutrition, infections, perinatal asphyxia, iron deficiency, lead toxicity and poverty are detrimental for cognitive development in children^3–5^. Children from poorer households are more likely to experience unsupportive parenting, lack of caregiver education, more stressful events, lack of stimulating environment including play materials and poor education, which in turn result in poor cognitive development and scholastic performance. This compromise in education and subsequent earning capacity in these children further produces an inter-generational transmission of poverty^2^. On the other hand, positive and stimulating environment prevailing at home and appropriate learning opportunities during preschool years can augment a child’s developmental and cognitive abilities^6^.

Global evidence suggests that early childhood education (ECE) offered at a better quality can positively impact a child’s intellectual, social and emotional development, and continued learning in future^7,8^. In a Brazilian cohort, children who received structured ECE, compared to those who did not, had higher cognition scores by 8 units at 5 years of age, after adjusting for home environment and socioeconomic status^6^. Similarly, ECE programmes in low-and-middle income country (LMIC) settings such as in Bangladesh have promoted academic achievements in mathematics, writing and reading domains for children in their primary grades^9^. In a preschool environment, in addition to direct learning opportunities in a structured manner, children can also have peer social interactions which can aid their indirect learning experiences. Thus, a centre-based education for pre-schoolers can help to attain better motor coordination, arithmetic skills, memory and concentration in children and may override many adverse childhood experiences^6,10,11^.

In India, the National Early Childhood Care and Education (ECCE) policy recommends that children between 3 and 6 years receive early education and care from Anganwadi centres, which are community-based^12–14^. Multi-age grouping is followed in which students of different ages and identified age levels are grouped in a single classroom to provide effective instruction. The medium of interaction in the ECCE centre should be the home language or mother tongue. In the early years, the focus would be on listening and speaking activities, facilitated through free play with peers. In the region where the study was done, the ECCE Programme in Tamil language was conducted 5 days a week and for a minimum of 4 h duration,

The ECE programs typically aim to enhance intellectual and social abilities of children which can form a fulcrum for their subsequent learning development. They had considerable positive short-term effects and somewhat smaller long-term effects on cognitive development more so for children from socio-economically disadvantaged families. Studies have shown that sustained high-quality early care and education can mitigate the consequences of poverty into adulthood^15,16^. Nevertheless, some studies and an international review highlight sub-optimal evidence of long-term effects of ECE^17^. Possible reasons for even a school-level ‘fade-out’ of ECE could be the quality of ECE, the absence of sustaining environments later and differing skillsets for learning in different age groups^18^. Another commonly cited explanation of the ‘fade-out’ effect of ECE programs on early academic skills is that it changes with time, and the gains often disappear after children transition to elementary school^15,18^. Thus, it is important to understand if structured ECE interventions have a long-term effect on cognition in the later childhood period, as this could help policymakers assess cost–benefit aspects better. In this context, the current study evaluates the association between structured ECE and cognition at 5 and 9 years of age in a birth cohort in Vellore, India. For this study, structured ECE was defined as specific structured language and learning inputs from early childhood curriculum and was provided in this area by both private and government aided schools in preschool settings. It is hypothesised that structured ECE will have a positive association with both 5- and 9 year cognition.

## Methods

This paper presents the analysis of a birth cohort follow-up, as part of a multi-country cohort study done in eight LMICs evaluating the role of enteric infections in early life on growth and development in children (‘The Etiology, Risk Factors and Interactions of Enteric Infections and Malnutrition and the Consequences for Child Health and Development (MAL-ED) Network Cohort)^19^. The study site in Vellore, India recruited 251 newborns between 2010 and 2012 from dense urban slum settlements following parental consent^20^. Children were intensively followed up through home visits till two years of age to capture information on sociodemographic details, morbidity status, anthropometry, and dietary intake, with subsequent follow-up visits made at 3, 4, 5, 7 and 9 years of age. This study was approved by the Institutional Review Board, Christian Medical College, Vellore for initial recruitment as well as follow-up visits (IRB 6769 for the original cohort and follow-up, IRB 11821 for the 9 year follow-up) and was conducted following the guidelines laid by World Medical Association Declaration of Helsinki. Before each recruitment, we obtained parental informed consent in written format and for the 9-year follow-up, additional child assent.

### Exposure variables

To evaluate child’s stunting status, trained Field research assistants (FRA) carried out standardized anthropometry measures using an infantometer for recording the length up to 2 years, and a stadiometer thereafter, till 9 years of age to the nearest cm. Z scores for length/height measurements were calculated based on Multicentre Growth Reference Study standards and children were classified as stunted at each time point if Z scores were below − 2 SD. Maternal intelligence quotient (IQ) raw scores were assessed using Raven’s Progressive Matrices by a single, trained psychologist. The Home Observation for the Measurement of the Environment (HOME) scale was administered by a trained social worker at 2 years to measure overall support received by the child at home from caregivers. At 5 years, caregivers were contacted for information about the number of months of structured ECE received by their children. The socioeconomic status (SES) assessment was based on the Water and Sanitation, Assets, Maternal education, and Income (WAMI) scores.

### Outcome measures

A single, trained psychologist conducted cognition evaluation for children in the community study clinic using Weschler’s Preschool Primary Scale of Intelligence (WPPSI) and Malin’s intelligence scale for Indian children (MISIC) at 5 and 9 years respectively^3,21^. The WPPSI scale was translated, and pilot-tested in local settings to assess cognition under verbal, performance, and processing speed domains. The MISIC scale which was adapted for use in the Indian setting from the original Wechsler scale, evaluated cognition under verbal and performance domains. Raw scores obtained by children under each domain were converted into IQ scores for analysis.

### Statistical analysis

Data was collected in paper forms and entered in the double-entry database system maintained by the central Data coordination centre of the MAL-ED study for the initial part of the birth cohort study. Descriptive statistics were used to summarize sex, SES and early childhood stunting status in percentages. Children living in households with WAMI scores ≥ 33rd percentile were classified as those belonging to relatively high SES. Stunting status of children at 2 and 5 years was used to group children into those who were never stunted, stunted at 2 years but recovered by 5 years, and, persistently stunted at 2 and 5 years. Based on tertile scores of structured ECE attendance expressed in months, children were classified into three groups. Children in group 1 had no exposure to structured ECE, whereas those in groups 2 and 3 attended 1–17 and 18–24 months, respectively in a structured preschool. HOME scores were summarized as median and interquartile (IQR) scores. Child’s IQ scores under each domain were presented as median (IQR) and box plots were used to visualize relationship between IQ scores, and groups based on preschool and structured preschool attendance. Correlation co-efficients were computed to check for correlation between domain scores of cognition measures at 5 and 9 years. To measure the association between structured ECE and IQ scores, bivariate and multivariate regression analyses were performed. The normality of dependent variables was assessed using the Shapiro–Wilk test. Linear regression was performed only for performance IQ (PIQ) at 5 years, while quantile regression was used to fit other domain scores such as verbal IQ (VIQ), processing speed IQ (PSIQ) and full-scale IQ (FSIQ) since normality assumptions for linear regression analysis were not met. Similarly, the predictor model for VIQ at 9 years was based on linear regression, while PIQ and total IQ scores at 9 years were modelled using quantile regression. Multivariable models included independent variables that were statistically significant in bivariate analysis at p < 0.05, and collinearity between the variables was analysed using correlation statistics. R^2^ values and the Hosmer–Lemeshow goodness-of-fit test were considered for assessing model fitness in the case of linear regression models. Beta coefficients along with 95% confidence intervals (CI) were reported and a p-value less than 0.05 was considered as statistical significance. Statistical analysis was performed using STATA version 14 (Stata Statistical Software: Release 14, StataCorp LP, College Station, TX).

### Ethics approval

This study was approved by the Institutional Review Board, Christian Medical College, Vellore for initial recruitment as well as follow-up visits (IRB 6769 for the original cohort and follow-up; IRB 11821 for the 9 year follow-up). Participants were enrolled after due written informed consent before inclusion in the study. This research was conducted ethically following the World Medical Association Declaration of Helsinki.

## Results

A cohort of 251 newborns were recruited as part of the Vellore cohort of MAL-ED by screening 301 pregnant women from the study area between 2010 and 2012. At recruitment, a female preponderance (55%) was observed in the cohort. About 212 (84.46%) and 205 (81.67%) children were available for follow-up at 5 and 9 years^21^ and the most common reason for non-participation was migration of the family outside study area. The 5-year recruitment was conducted between 2015 February and 2017 February. The 9 year recruitment was planned between 2019 February and 2021 February. There was a disruption in recruitment for 6–7 months in 2020 due to the Covid-19 pandemic. We completed cognitive and other clinic-based assessments for all children by April 2021.

There was no significant difference in cohort characteristics such as sex and socioeconomic status between the follow-up time points (Table 1). Of 212 children followed up at 5 years, 41 (19.34%) were stunted at two years but recovered by 5 years, whereas 58 (27.36%) children were found to be stunted both at two and five years. Proportion of children who attended structured ECE was 54.25% with median months of attendance 8 months.

**Table 1:** Sociodemographic characteristics of children in Vellore cohort of MAL-ED.

Variables	Enrolment (n = 251) %	5 years (n = 212) %	9 years (n = 205) %
Sex			
Male	113 (45.02)	98 (46.23)	96 (46.83)
Female	138 (54.98)	114 (53.77)	109 (53.17)
Socioeconomic status (SES)*			
Low (WAMI < 33rd percentile)	71 (30.2)	65 (30.66)	–
Relatively high (WAMI ≥ 33rd percentile)	164 (69.79)	147 (69.34)	–
Median (IQR) raw scores of maternal cognition	45 (36–51)		
Median (IQR) HOME scores at 2 years	41 (38–43)		
Median (IQR) months of structured ECE	–	8 (0–18)	–
Early childhood stunting			
Never stunted at 2 and 5 years	–	113 (53.30)	–
Stunted at 2 years but recovered at 5 years	–	41 (19.34)	–
Stunted at both 2 and 5 years	–	58 (27.36)	–

Sex and SES characteristics did not differ significantly across baseline, 5 and/or 9 years of follow-up.

*MAL-ED*. The etiology, risk factors and interactions of enteric infections and malnutrition and the consequences for child health and development study, *IQ* intelligence quotient, *IQR* interquartile range.

(P value > 0.05).

*SES assessment at baseline was available only for 235 children.

**Figure 1:**
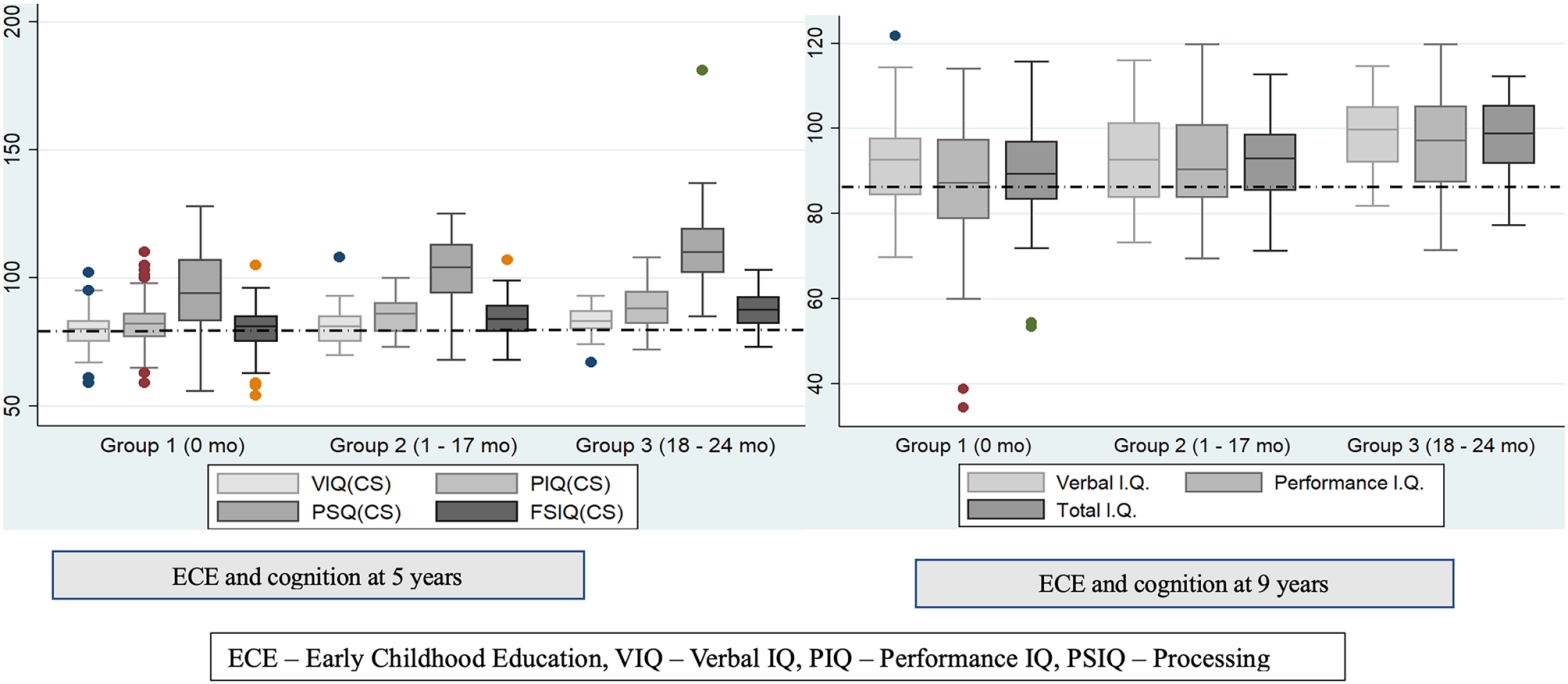
Comparison of cognition scores across tertile groups based on ECE attendance in the MAL-ED cohort.

Median (IQR) WPPSI IQ scores measured at 5 years under verbal, performance, processing speed and full-scale domain were 81 (77–85), 84 (79–90), 103 (88–113) and 84 (79–89) respectively (Fig. 1). Bivariate analysis of cognition scores across tertile categories of structured ECE showed that children belonging to the highest tertile (18–24 months of attendance) had significantly higher IQ scores by 3, 6.15, 16 and 7 units in VIQ, PIQ, PSIQ and FSIQ domains respectively at 5 years, compared to those in the lowest tertile (nil attendance to structured preschool) (Table 2). Further, multivariate analysis accounting for maternal cognition, HOME scores, SES and early life stunting status showed that structured ECE between 18 and 24 months (highest tertile) was significantly associated with higher cognition scores in children at 5 years, with highest increase in PSIQ domain [β: 19.55 (11.26–27.77)], followed by FSIQ [β: 6.75 (2.96–10.55)], PIQ [β: 5.54 (2.11–8.98)] and VIQ domains [β: 3.32 (0.74–5.91)], compared to those who did not attend structured ECE (lowest tertile). It is notable that children in the second tertile of structured ECE with a median duration of 1–17 months also had higher PIQ and PSIQ in the bivariate analysis compared to those in the lowest tertile (nil attendance), however, this association was not found to be statistically significant in the multivariate analysis (Table 3). A sensitivity analysis was conducted categorizing the duration of structured ECE under two different scenarios to support the main analysis based on tertile categorization of ECE exposure. In the first scenario, structured ECE duration was categorized year-wise as per the existing education curriculum in India into ‘no attendance’, ‘1–12 months’ and ‘13–24 months’. Children exposed to 13–24 months of ECE had a significant increase in FSIQ by 4.8 compared to those who did not attend structured ECE [β: 4.82 (2.1–7.5)] in multivariate analysis. In the second scenario, structured ECE duration was divided as quartiles into ‘no attendance’, ‘1–8 months’, ‘8–18 months’ and ‘ > 18 months’ of exposure and included in multivariate analysis. Compared to children who did not have structured ECE, those exposed to ‘8–18 months’ and ‘ > 18 months’ had a significant increase in FSIQ scores by 3.4 [β: 3.4 (0.5–6.3)] and 6.4 [β: 6.4 (3.2–9.6)], respectively (data not shown).

**Table 2:** Bivariate analysis measuring factors associated with cognition scores of children in MAL-ED cohort at 5 years (N = 212).

Characteristics	Verbal IQ (VIQ) at 5 years		Performance IQ (PIQ) at 5 years		Processing speed IQ (PSIQ) at 5 years		Full scale IQ (FSIQ) at 5 years	
	Beta co-efficient (95% CI)	P value	Beta co-efficient (95% CI)	P value	Beta co-efficient (95% CI)	P value	Beta co-efficient (95% CI)	P value
Groups based on ECE								
Group 1 (nil attendance)	*Ref*		*Ref*		*Ref*		*Ref*	
Group 2 (1–17 months)	1 (− 1.07–3.07)	0.343	**3.35 (0.45–6.25)**	**0.024**	**10 (2.11–17.89)**	**0.013**	3 (− 0.54–6.54)	0.097
Group 3 (18–24 months)	**3 (0.98–5.02)**	**0.004**	**6.15 (3.33–8.97)**	** < 0.001**	**16 (8.34–23.68)**	** < 0.0001**	**7 (3.55–10.45)**	** < 0.001**
Female	0 (− 1.63–1.63)	1.000	0.42 (− 2.05–2.88)	0.740	4 (− 2.67–10.67)	0.238	**3 (0.47–5.53)**	**0.020**
Maternal cognition raw scores	**0.13 (0.04–0.22)**	**0.006**	**0.18 (0.07–0.30)**	**0.002**	**0.44 (0.16–0.73)**	**0.003**	**0.22 (0.11–0.34)**	** < 0.001**
Relatively high SES at 5 years (WAMI ≥ 33rd percentile)	1 (− 0.63–2.63)	0.227	**3.74 (1.13–6.36)**	**0.005**	**10 (3.73–16.27)**	**0.002**	3 (− 0.43–6.43)	0.086
HOME inventory scores at 2 years	0.2 (-0.05–0.45)	0.114	**0.96 (0.63–1.30)**	** < 0.0001**	**1.43 (0.55–2.30)**	**0.002**	**0.67 (0.26–1.07)**	**0.001**
Stunting status in early life								
Never stunted	*Ref*		*Ref*		*Ref*		*Ref*	
Stunted at 2 years but recovered at 5 years	0 (− 2.02–2.02)	1.000	0.70 (− 2.56–3.95)	0.673	2 (− 6.26–10.26)	0.634	1 (− 2.45–4.45)	0.569
Stunted at 2 and 5 years	1 (− 2.79–0.79)	0.272	− 1.51 (− 5.15–2.13)	0.414	− 5 (− 14.25–4.25)	0.288	− 3 (− 6.86–0.86)	0.127

Quantile regression was carried out for VIQ, PSIQ and FSIQ scores, and linear regression was carried out for PIQ scores at 5 years.

*MAL-ED*. The etiology, risk factors and interactions of enteric infections and malnutrition and the consequences for child health and development study, *IQ* intelligence quotient, *SES* socioeconomic status.

Significant values are in bold.

**Table 3:** Multivariate analysis measuring association between structured ECE and cognition scores of children at 5 years (N = 212).

Characteristics	Verbal IQ (VIQ) at 5 years		Performance IQ (PIQ) at 5 years		Processing speed IQ (PSIQ) at 5 years		Full scale IQ (FSIQ) at 5 years	
	Beta co-efficient (95% CI)	P value	Beta co-efficient (95% CI)	P value	Beta co-efficient (95% CI)	P value	Beta co-efficient (95% CI)	P value
Groups based on ECE								
Group 1 (nil attendance)	*Ref*		*Ref*		*Ref*		*Ref*	
Group 2 (1–17 months)	0.66 (− 1.82–3.15)	0.598	2.97 (− 0.17–6.11)	0.064	7.33 (− 0.24–14.90)	0.058	3.13 (− 0.33–6.60)	0.076
Group 3 (18–24 months)	**3.32 (0.74–5.91)**	**0.012**	**5.54 (2.11–8.98)**	**0.002**	**19.55 (11.26–27.77)**	** < 0.0001**	**6.75 (2.96–10.55)**	**0.001**
Female	1.20 (− 0.75–3.15)	0.226	0.65 (− 1.75–3.05)	0.592	**6.71 (1.07–12.35)**	**0.020**	**3.38 (0.69–6.07)**	**0.014**
Maternal cognition raw scores	**0.13 (0.03–0.22)**	**0.009**	**0.14 (0.02–0.25)**	**0.019**	0.21 (− 0.07–0.48)	0.140	**0.16 (0.04–0.29)**	**0.013**
Relatively high SES at 5 years (WAMI ≥ 33rd percentile)	-2.11 (− 4.44–0.23)	0.076	1.20 (− 1.66–4.06)	0.408	3.12 (− 3.68–9.92)	0.367	0.41 (− 2.76–3.59)	0.798
HOME inventory scores at 2 years	0.24 (− 0.08–0.56)	0.148	–		0 (− 0.91–0.91)	0.992	−	
Stunting status in early life								
Never stunted	−		−		*Ref*		*Ref*	
Stunted at 2 years but recovered at 5 years					− 6.56 (–13.93–0.77)	0.079	− 0.54 (− 4.04–2.60)	0.761
Stunted at 2 and 5 years					− 3.75 (–10.48–2.98)	0.273	− 1.27 (− 4.47–1.94)	0.437

Quantile regression was carried out for VIQ, PSIQ and FSIQ scores, and linear regression was carried out for PIQ scores at 5 years.

*ECE* Early childhood education, *MAL-ED*. The etiology, risk factors and interactions of enteric infections and malnutrition and the consequences for child health and development study, *IQ* intelligence quotient and *SES* socioeconomic status.

Significant values are in bold.

Median (IQR) cognition scores measured at 9 years under verbal, performance, and total IQ scores were 94.4 (87.2–100.6), 91.6 (82.9–101.2) and 93.4 (85.5–100.2) respectively. Correlation coefficients between verbal IQ, performance IQ and total scores IQ at 5 and 9 years were 0.6, 0.6 and 0.7 respectively (data not shown). Exposure to structured ECE showed a positive association with VIQ, PIQ and total IQ scores at 9 years. In bivariate analysis, children in the highest tertile category (18–24 months) of structured preschool attendance showed about 7–10 points increase in IQ scores across various domains of cognition, with effect size being highest in the PIQ domain compared to those who did not attend structured preschool (Table 4). This association remained significant in the multivariate model, with children in the highest tertile category scoring higher IQ scores than those in the lowest tertile, after adjusting for maternal IQ, SES and early-life stunting. Structured preschool exposure for about 18–24 months had the greatest impact on PIQ domain with a higher effect size [β: 8.82 (2.60–15.03)], followed by total IQ [β: 7.24 (2.52–11.90)] and verbal IQ domains [β: 5.25 (1.88–8.62)], compared to those without structured preschool attendance (Table 5). Similar to 5 years analysis, a sensitivity analysis was conducted categorizing the duration of structured ECE under two different scenarios. In the first scenario, where structured ECE duration was categorized year-wise into ‘no attendance’, ‘1–12 months’ and ‘13–24 months’, children exposed to 13–24 months of ECE had an increase in FSIQ by 4.5 compared to those who did not attend structured ECE [β: 4.5 (0.3–8.6)] in multivariate analysis. In the second scenario, where structured ECE duration was divided into quartiles, it was observed that children with structured ECE of ‘ > 18–24 months’ had a significant increase in FSIQ scores by 7.7 compared to those who did not have any exposure to ECE [β: 7.8 (3.0–12.4)] (data not shown).

**Table 4:** Bivariate analysis measuring factors associated with cognition scores of children at 9 years (N = 203).

Characteristics	Verbal IQ (VIQ) at 9 years		Performance IQ (PIQ) at 9 years		Total IQ at 9 years	
	Beta co-efficient (95% CI)	P value	Beta co-efficient (95% CI)	P value	Beta co-efficient (95% CI)	P value
Groups based on structured ECE attendance						
Group 1 (nil attendance)	*Ref*		*Ref*		*Ref*	
Group 2 (1–17 months)	1.53 (− 1.62–4.67)	0.340	3.20 (− 2.34–8.74)	0.256	3.70 (− 0.92–8.32)	0.116
Group 3 (18–24 months)	**7.17 (4.10–10.25)**	** < 0.0001**	**10 (4.59–15.41)**	** < 0.0001**	**9.60 (5.08–14.12)**	** < 0.0001**
Female	− 0.20 (− 2.91–2.51)	0.885	− 1 (− 6.27–4.27)	0.709	− 1 (− 5.01–3.01)	0.623
Maternal cognition raw scores	**0.26 (0.14–0.38)**	** < 0.0001**	**0.42 (0.21–0.64)**	** < 0.0001**	**0.34 (0.16–0.52)**	** < 0.0001**
Relatively high SES at 5 years (WAMI ≥ 33rd percentile)	2.71 (− 0.18–5.60)	0.066	4.80 (− 0.32–9.92)	0.066	**4.60 (0.31–8.89)**	**0.036**
HOME inventory scores at 2 years	**0.75 (0.37–1.13)**	** < 0.0001**	**0.95 (0.21–1.69)**	**0.012**	**1.02 (0.48–1.55)**	** < 0.0001**
Stunting status in early life						
Never stunted	*Ref*		*Ref*		*Ref*	
Stunted at 2 years but recovered at 5 years	− 0.12 (− 3.73–3.49)	0.947	6 (− 0.30–12.30)	0.062	3.10 (− 1.58–7.78)	0.193
Stunted at 2 and 5 years	− **4.69 (**− **8.70**– − **0.69)**	**0.022**	0.60 (− 6.39–7.59)	0.866	− 3.30 (− 8.49–1.89)	0.211

Quantile regression was carried out for PIQ and total IQ scores, and linear regression was carried out for VIQ scores at 9 years.

*ECE* early childhood education, *MAL-ED*. The etiology, risk factors and interactions of enteric infections and malnutrition and the consequences for child health and development study, *IQ* intelligence quotient, *SES* Socioeconomic status.

Significant values are in bold.

**Table 5:** Multivariate analysis measuring association between structured ECE and cognition scores of children at 9 years (N = 203).

Characteristics	Verbal IQ (VIQ) at 9 years		Performance IQ (PIQ) at 9 years		Total IQ at 9 years	
	Beta co-efficient (95% CI)	P value	Beta co-efficient (95% CI)	P value	Beta co-efficient (95% CI)	P value
Groups based on structured ECE attendance						
Group 1 (nil attendance)	*Ref*		*Ref*		*Ref*	
Group 2 (1–17 months)	0.04 (− 3.14–3.23 )	0.979	1.08 (− 4.79–6.96)	0.717	2.44 (− 2.02 – 6.90)	0.282
Group 3 (18–24 months)	**5.25 (1.88–8.62)**	**0.002**	**8.82 (2.60–15.03)**	**0.006**	**7.24 (2.52–11.96)**	**0.003**
Female	− 0.50 (− 3.06–2.07)	0.703	− 4.65 (− 9.38– − 0.08)	0.054	− 1.96 (− 5.56–1.63)	0.283
Maternal cognition raw scores	**0.20 (0.08–0.33)**	**0.002**	**0.33 (0.11–0.56)**	**0.004**	**0.30 (0.13–0.47)**	**0.001**
Relatively high SES at 5 years (WAMI > = 33rd percentile)	− 2.01 (−5.04–1.03)	0.194	0.07 (− 5.53–5.67)	0.981	0.05 (− 4.21–4.30)	0.982
HOME inventory scores at 2 years	**0.47 (0.05–0.89)**	**0.028**	0.07 (− 0.70–0.84)	0.864	0.29 (− 0.30–0.87)	0.334
Stunting status in early life						
Never stunted	*Ref*		*Ref*		*Ref*	
Stunted at 2 years but recovered at 5 years	− 0.21 (− 3.61–3.18)	0.901	− 5.15 (− 11.40–1.10)	0.106	− 2.92 (− 7.67–1.83)	0.123
Stunted at 2 and 5 years	− **3.38 (**− **6.41–** − **0.36)**	**0.029**	− 1.30 (− 6.88–4.28)	0.647	− 2.63 (− 6.87–1.61)	0.223

Quantile regression was carried out for PIQ and total IQ scores, and linear regression was carried out for VIQ scores at 9 years.

*ECE* early childhood education, *MAL-ED*. The etiology, risk factors and interactions of enteric infections and malnutrition and the consequences for child health and development study, *IQ* intelligence quotient, *SES* Socioeconomic status.

Significant values are in bold.

## Discussion

This prospective follow-up study done among children living in an urban Indian slum setting evaluated the association between structured ECE and cognition scores at 5 and 9 years of life. Attending structured ECE was associated with higher cognition scores by 3–19 points at 5 years, with the highest increase seen in the processing speed domain of cognition, despite corrections with maternal cognition, SES, home factors, and early childhood stunting, compared to those who did not attend. This association between structured ECE and cognition remained significant even in later childhood, where an increase in IQ scores by 5–9 points at 9 years was observed after correcting for SES, maternal cognition, home factors, and childhood stunting status.

Global evidence states that early childhood adversities, particularly, poor home environment negatively impact cognitive abilities during childhood and continue to impact even in adult life^22^. In the current birth cohort studied in Vellore, a nurturing home environment in early childhood increased developmental cognition scores, while responsive caregiving resulted in augmented language development as evidenced by a previous publication from the same cohort^23^. It should also be highlighted that developmental trend analysis of this LMIC birth cohort showed a decline in overall developmental scores including that of cognition and language between 6 and 36 months of age, owing to SES, home environments, childhood stunting and low blood iron status^23^. A similar association was reported from a Brazilian cohort where children from healthier home environments had better cognition scores by 5 points in the fifth year of follow-up^6^.

The negative impacts that early childhood adversities have on cognition can be mitigated by appropriate psychosocial stimulation as reported in the same Brazilian cohort, where children attending nursery classes had higher cognition scores by 8 points at follow-up^6^. The present study concurs with this finding where structured ECE was associated with higher full-scale IQ scores by 7 units at 5 years. Another important finding of this study is the sustenance of this positive impact on cognition even at 9 years of age. This finding is in line with a Uruguay study which showed that preschool education had a positive impact on academic achievements both at the time of entering primary school and six years later^24^. Similar persisting effects of good quality ECE were reported from Abecedarian low-income cohort and the ‘1991 NICHD study of early childcare and youth development’, where ECE was associated with significant life success benefits as evidenced by additional years of education, better employment and vocational opportunities, and better wages^15,16^.

Preschool years represent a crucial period for the development of cognitive, linguistic, social, and psychomotor competencies in children. Early childhood care and education help to overcome environmental adversities due to impoverishment since the plasticity of the developing brain allows the reorganisation of brain circuits in response to psychosocial stimulation^8,25^. Another plausibility for the impact of centre-based early childhood interventions on academic achievements in later life would be based on the ‘cognitive advantage hypothesis’ where children from impoverished settings were able to capitalize the cognitive gains acquired due to interventions into academic achievements later in their life^15^. Aptly, the 75th World Health Assembly included opportunities for early learning in the Nurturing Care Framework to optimise early child development^26^. The current paper adds further evidence to this in terms of the need for a structured learning opportunity in early childhood.

In line with WHO and UNESCO’s recommendation of strengthening ECE as a cost-effective strategy for a country to make sustainable progress, the preschool education programme under the Integrated Child Development Services (ICDS) scheme has percolated in length and breadth of the country reaching out to tens of millions of children in India^8,27–30^.A co-prioritisation of preschool education, along with nutritional rehabilitation, has been highlighted by recent government initiatives and is in the right direction. A study from Maharashtra during 2004–05, introduced an ECE package in Anganwadis which included refresher training sessions to Anganwadi worker and financial support to purchase play materials. This intervention was found helpful in increasing IQ scores by 10 points in children compared to controls and is in concurrence with our findings^31^. Recent evidence from Tamil Nadu demonstrated the effectiveness of introducing an extra worker exclusively concentrating on ECE within the existing Anganwadi system, which resulted in exclusive ECE opportunities and an increase in cognition scores in mathematics, language and executive domains was noted in these children at 18 months, post-intervention^12^. Different methods to provide structured educational inputs in early childhood along with nutritional measures within the ICDS system can be explored by further studies in other Indian states to aid policy decisions for the country.

Genetic influences of cognitive abilities cannot be overlooked. In our current analysis as well as published literature from the same cohort, maternal cognitive capacity was shown to have a significant association not only with child development, and cognitive abilities but also with early childhood home environment^3,21,23,25^. Our analyses in the present study were corrected for maternal cognition recognising this genetic leverage.

There are many limitations to the current analysis. The cohort had a comparatively small sample size. We should also keep in mind that the Wechsler scale used at 5 years of age was not completely adapted for Indian settings. Both cognitive assessment measures used in the current analysis evaluate logical intelligence, but not other components of intelligence including but not limited to emotional intelligence, visuospatial intelligence, musical intelligence, etc. Quality of preschool and primary school education variables were not available for consideration in this study as well. Strengths of the study include minimal loss to follow-up, availability of good quality of early childhood data and India-specific cognitive analysis at 9 years of age.

## Conclusions

This cohort study done in an impoverished urban Indian setting has brought out the positive association of attending structured ECE on school entry cognition at 5 years and mid-childhood cognition at 9 years of life. Strengthening ECE through a multi-pronged approach of structured preschool curriculum, provision of adequate play materials, providing trained manpower and enabling centres with information and communication tools could facilitate to maximize the cognitive potential of human capital in India.

## Data Availability

MAL-ED dataset till 5 years of age is uploaded on www.clinepidb.org. Further dataset can be shared by the Corresponding Author, on reasonable request.
